# Kinetics and Mechanism of Selenium(IV) Oxidation by Aqueous Bromine Solution

**DOI:** 10.1021/acsomega.3c01497

**Published:** 2023-04-21

**Authors:** György Csekő, Boglárka Nyitrai, Attila K. Horváth

**Affiliations:** Department of General and Inorganic Chemistry, Faculty of Sciences, University of Pécs, Ifjúság útja 6, Pécs H-7624, Hungary

## Abstract

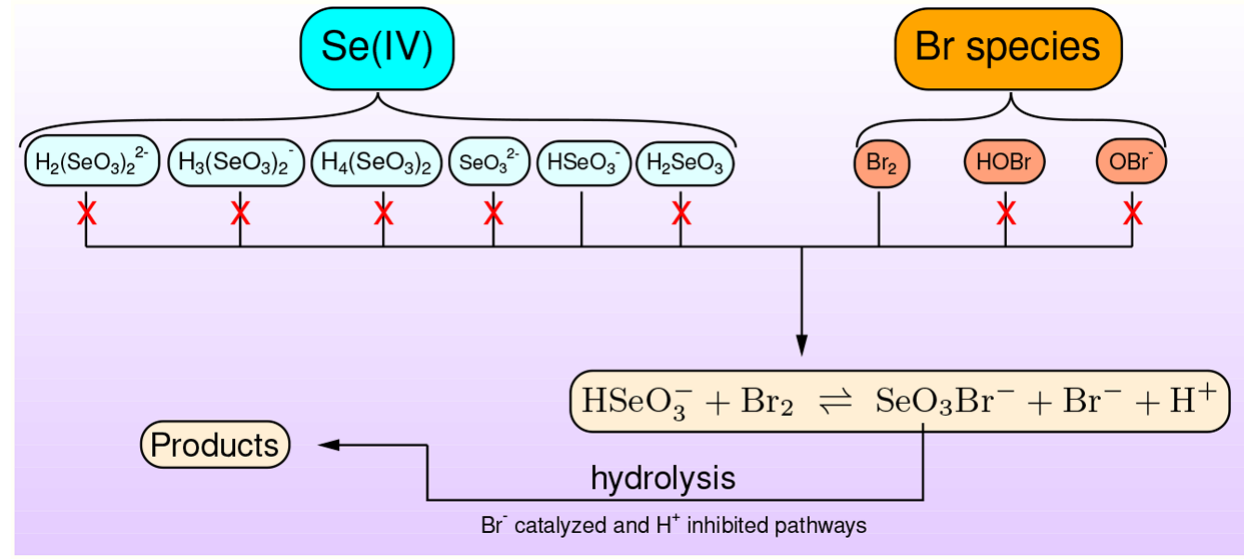

The bromine–selenite reaction at strongly acidic conditions was investigated by monitoring the absorbance–time traces at the isosbestic point of the bromine–tribromide system at a constant ionic strength (0.5 M adjusted by sodium perchlorate) and temperature. Despite the simplicity of the stoichiometry, the kinetics was found to be very complex. Although the formal kinetic orders of the reactants bromine and selenite are strictly 1, that of the hydrogen ion varies from −2 to less than −3 and notably depends on the initial bromide concentration as well. The bromide ion also inhibits the reaction, making the whole system as a sound example of efficient autoinhibition. We have clearly shown that the inhibitory effect of the bromide ion cannot be explained quantitatively by either exclusively considering the unreactivity of the tribromide ion over elemental bromine or driving the reaction via hypobromous acid formed from the well-known hydrolysis of bromine in aqueous solutions. Instead of that, bromonium ion transfer initiating equilibrium is suggested between the selenium(IV) and bromine species to produce bromide ion and SeO_3_Br^–^ followed by the hydrolysis of this short-lived intermediate. This hydrolytic transformation was found to be catalytic with respect to hydroxide and bromide ions as well. We have also demonstrated that, among the wide variety of selenium species present in the acidic aqueous solution, the best result can be obtained by considering HSeO_3_^–^ as the kinetically active species toward bromine. The proposed mechanism containing 10 acid–base equilibria with known equilibrium constants, the above-mentioned initiating equilibrium, and the hydrolysis of SeO_3_Br^–^ is able to fit all 49 kinetic absorbance–traces simultaneously, taking into account properly the most important characteristics of the measured data at strongly acidic conditions. Furthermore, this kinetic model was further extended by the direct reactions of hypobromous acid with selenium(IV) species suggested previously with reasonably modified rate coefficients to describe the pH dependence of the apparent second-order rate coefficients over the pH = 1–13 range, providing a useful tool to predict more accurately the kinetic behavior of selenium(IV) species in water treatment process conditions.

## Introduction

Selenium is a versatile element that exists in various oxidation states in different inorganic and organic substances. These compounds may play a substantial role in different areas of life sciences, such as plant biology, living organisms, and environmental studies of groundwaters. For instance, it has recently been shown that moderate selenite treatment of soils positively enhanced the photosynthesis of rice plants, thus increasing the yield of rice.^1^ In addition to that, the survival probability of cadmium polluted rice plants is significantly increased upon selenite solution treatment.^2^ Even though further investigations are required to elucidate completely the mechanistic details of the effect of selenite solution, it seems that selenite might act as an oxidative stress-decreasing factor in rice plants. In contrast to that, selenite may also be harmful when reaching a critical level in fresh waters^3^ or in soils^4,5^ due to a severe accumulation process. Besides, selenium is also an important trace element for human beings, essential for fine functioning of many enzymes in relevant biochemical processes, but the range between the dietary deficiency, treatment efficiency, and toxicity might be quite narrow. Up to 0.3 mass %, it induces the apoptosis of malignant cells, but above that level it may directly lead to healthy cell death.^6^ The proposed daily dose of selenium for a healthy adult was found to be 55 μg^7^ necessary for the buildup of essential selenoproteins. In addition, research clearly showed that an additional portion of 140–200–300 μg of selenium uptake^7,8^ has a preventive effect in the case of lung cancer,^7^ prostate cancer,^9^ and osteosarcoma^6^ or helps to recover the insufficient functioning of reproductive organs.^10^ At the same time, selenium(IV) may behave as an antioxidant^11^ and a prooxidant;^12^ thus this duality may also play a crucial role not only in the prevention but also in the cancer treatment procedures as well.^13^ Therefore, it seems to be of special importance to unravel the mechanistic details of those reactions in aqueous solutions, where selenium-containing species are involved. It is evident that an adequate and reliable answer could only be expected to these problems if the mechanistic details of the reactions of selenium species in aqueous conditions would be well-elucidated. Surprisingly, however, there is limited information available in the literature about the redox transition of selenite ion. One possible explanation of this fact is that aqueous selenite solution may contain multiple species in commensurable amounts, from which it is not easy to find the kinetically active one. It is well-known that depending on the pH and the concentration selenium(IV) may be found in various forms in aqueous conditions.^14,15^ At low total selenite concentration, depending on the pH, SeO_3_^2–^, HSeO_3_^–^, and H_2_SeO_3_ may be present overwhelmingly in the solution, but at a higher total concentration (even already around 0.01 M), the dimerization of differently protonated forms of selenium(IV) might also occur^16^ providing a straightforward possibility for all species involved to be kinetically active reactants. Our preliminary experiments in the selenite–bromate system have revealed that the formation of bromine during the course of this reaction is somewhat delayed and its characteristic sigmoidal-shaped profiles suggested an appearance of the autocatalysis-driven clock reaction^17^ meaning that the rate of the reaction between the clock species (bromine) and selenite should be commeasurable with that of the formation of bromine from the bromide–bromate reaction. It straightforwardly means that the title reaction could easily be followed by conventional UV–vis spectroscopy providing a promising possibility to determine its apparently complex kinetics and mechanism. A survey of the literature has revealed that the title reaction was first studied by Dikshitulu and Babu^18^ using the in situ generation of bromine from *N*-bromosuccinimide and bromide ion. They have found that the rate of the reaction is retarded by hydrogen and bromide ions as well, and both the monomeric and the dimeric forms of selenite can be treated as reactive species toward bromine, with the latter one being more reactive. The inhibitory effect of bromide ion was interpreted by the decreased reactivity of Br_3_^–^ as compared to that of bromine. Our recent series studying the kinetics of the oxidation of iodine with various sulfur-centered reducing agents, however, has clearly revealed that the formation of trihalide ion along with its decreased reactivity toward the reactants may only play a marginal role in the appearance of halide ion inhibition, if it plays a role at all.^19−24^ In all of these cases, it is rather the consequence of the initiating equilibrium of a halonium ion transfer process from the halogen to the sulfur-center of the substrate molecule yielding halide ion and a short-lived intermediate being responsible for this kinetic effect. On the basis of the sulfur–selenium chemical analogy, similar behavior seems to be soundly conceivable. Moreover, because halide ion is a product of these reactions, this phenomenon should rather be called autoinhibition. More recently, Liu et al. published a comprehensive study on the oxidation of selenite by various oxidants including bromine.^25^ Actually, not bromine but hypobromous acid was used as an oxidizing agent, although it is out of question that bromine was also present in their study because hypobromous acid was prepared upon oxidizing excess of bromide ion by hypochlorous acid.^25^ They have found that the following rate law is valid over a relatively wide pH range:
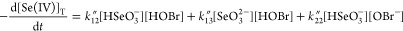
1where [Se(IV)]_T_ represents the total selenium(IV) concentration, and *k*_12_^″^, *k*_13_^″^, and *k*_22_^″^ were found to be 2300 ± 600, 74 000 ± 3000, and 820 ± 30 M^–1^ s^–1^, respectively. The main problem with this outcome is that *k*_13_^″^ and *k*_22_^″^ are kinetically indistinguishable from each other due to the rapidly established protonation and deprotonation equilibria of selenous acid and hypobromous acid. Because p  = 8.5 and p*K*_a,HOBr_ = 8.8,^26^ one may easily see that the last two terms of eq 1 may be reformulated as follows:
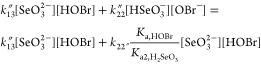
2Comparing the values of  = 411 ± 16 M^–1^ s^–1^ with *k*_13_^″^ = 74 000 ± 3000 M^–1^ s^–1^, it is at least questionable as to whether the second term of eq 2 may play any role at all in determining the kinetics of the given reaction. In addition to that inspection of Figure 2b published in page 4 of ref (25), one might easily notice that the given model is capable of sound description of measured *k*_app_ values (for definition, see eq 3 on page 3 of ref (25)) up to pH = 7, below which the deviation starts to increase significantly. The logarithmic scale in that figure reveals that the deviation between the measured and calculated *k*_app_ values may actually reach even a factor of 5. One possible reason for the increasing deviation as the pH decreases is that the direct reaction between bromine and selenite ion was not taken into consideration. This survey has clearly revealed that only quite limited and possibly improper information is available in the literature about this environmentally important reaction. We, therefore, report here a detailed kinetic and mechanistic investigation of this challenging system.

## Experimental Section

### Chemicals

All of the reagents, including sodium-selenite, sodium-perchlorate, sodium-dihydrogen phosphate, phosphoric acid, sodium bromide, and bromine, were analytical grade reagents and used without further purification. The stock solutions were prepared from twice ion-exchanged distilled water, which was further distilled twice atmospherically to remove any residues originating from the exchange resin and was also deoxygenated by bubbling through argon gas for at least 20 min. The buffer solutions were made from phosphoric acid, and its exact concentration was determined by classical titrimetry. The pH of the buffer solution was adjusted by addition of the calculated amount of sodium-dihydrogen phosphate by taking the p*K*_a1_ of phosphoric acid to be 1.84.^26^ The selenite and the bromide stock solutions were prepared by weighing the calculated amount of solid materials. The bromine stock solution was made from dissolving elemental bromine in 0.001 M perchloric acid solution to prevent the hydrolysis of bromine. Its concentration was checked by UV–vis spectroscopy at the isosbestic point of the bromine–tribromide system at 450 nm, where  =  = 104 M^–1^ s^–1^.^27^ The ionic strength of all solutions was adjusted to 0.5 M by addition of the necessary amount of sodium perchlorate.

### Instrumentation

The kinetic measurements were performed in a standard quartz cuvette equipped with a Teflon cup having an optical path of 1 cm. The Teflon cup was also sealed with Parafilm to minimize the escape of bromine. To maintain the solution homogeneously, magnetic stirrer bars were used (having a length of 8 mm and a diameter of 2 mm) in each cuvette. The reaction was monitored by a Zeiss S600 diode array spectrophotometer at the wavelength range of 400–900 nm, and every kinetic run contained at least 1000 absorbance–time data pairs originally. All of the solutions kept in the cell holder of the instrument were thermostated at 25.0 ± 0.1 °C.

### Kinetic Experiments

Altogether 49 kinetic experiments were performed at different initial concentrations. The concentration ranges used in these experiments were as follows: 0.49–4.9 mM, 1.1–6.8 mM, 0–31.2 mM, and 0.85–1.70 in the cases of selenite, bromine, bromide ion, and the pH, respectively. The solutions were delivered into the cuvette by the following order: first the perchlorate and the buffer solutions were introduced from an Eppendorf pipet, followed by the bromine solution. The measurements were initiated at that time to determine precisely the initial bromine concentration taking into consideration its volatility. After a well-defined time-point, finally the selenite solution was added to initiate the reaction from a fast-delivery pipet. All of the measurements were followed up to at least 95% stoichiometric conversion to gain complete information about the whole course of the reaction.

### Data Treatment and Evaluation

To avoid a time-consuming calculation, the number of the absorbance–time data pairs in each kinetic curves has been reduced approximately to 60 on the basis of the principle of equivalent arc length, the method that was described in detail elsewhere.^28^ The absorbance–time traces (measured at 450 nm where only Br_2_ and  absorb the light) obtained by this way were evaluated by the Chemmech program package developed for the determination of the kinetic parameters by minimizing the average absolute deviation between the measured and calculated data-pairs of all of the measurements taken into account simultaneously.^29^ The criterion of the best was to obtain not more than 0.004 absorbance unit, which is the experimentally achievable and reasonable limit of error of the absorbance measurements.

## Results and Discussion

### Equilibria of Selenium(IV) Species in Aqueous Solutions

The most comprehensive equilibrium study of selenium(IV) species was reported by Barcza and Sillén in 1971 at various media.^14^ The general reaction may be represented by
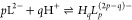
3where L^2–^ represents selenite ion () and the formation constant of H_*q*_(SeO_3_)_*p*_^(2*p*–*q*)–^ (mono- and binuclear) species (β_*pq*_) is defined as follows:
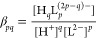
4Their formation constants at *I* = 1.0 M as well as at *I* = 0.3 M ionic strength in sodium perchlorate medium are summarized in the upper part of Table S1. Because our measurements were carried out in *I* = 0.5 M sodium perchlorate medium, we estimated the corresponding formation constants from Barcza and Sillén’s report.^14^ On the basis of these data, one can easily arrive at the following sequence of equilibria providing the correct distribution of selenium(IV) species present in the aqueous solution:
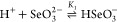
5
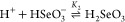
6
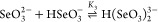
7
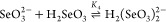
8
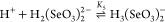
9
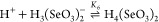
10The corresponding *K*_*i*_ values are also presented in Table S1, and these ones served as a starting point to adjust the forward and reverse rate constants for eqs 5–10 during the course of the fitting procedure in such a way that the given equilibrium is established rapidly. These equilibria along with their equilibrium constants reveal that under our experimental conditions (between the lowest and the highest total selenite concentrations) four selenium-containing species may be present in a notable amount (see Figure S1). Even though the monomeric forms are the major species under our experimental conditions meaning the relative amount of selenous acid (H_2_SeO_3_) and hydrogen-selenite (HSeO_3_^–^) varies 75–96% and 3–20%, especially at lower pH’s the dimeric forms (H_4_(SeO_3_)_2_ and ) also appear at appreciable amounts. At the highest total selenite concentration and at high acidity, the relative amount of H_4_(SeO_3_)_2_ can almost reach 7%; meanwhile, at the lowest acidity that of  may exceed 1%. Consequently, all of these species may be considered as potential candidates for being kinetically active reactants toward any bromine-containing species.

### Equilibria of Bromine Species in Aqueous Solution

The hydrolytic equilibrium of aqueous bromine solution has already been studied by several independent research groups,^30−32^ providing quite a diverse equilibrium constant of *K*_7_ = (0.67–12) × 10^–9^ M^2^ for the reaction given below:
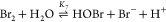
11A more recent study by Beckwith et al.^33^ has clarified that not just application of different ionic strengths may provide a significant change in *K*_7_ values, but eq 11 actually is the subject of a general acid–base assisted mechanism, meaning that the nature of acid applied also affects the kinetics. The study reported by Beckwith^33^ has provided the value of *K*_7_ = 6.06 × 10^–9^ M^2^ at an ionic strength of 0.5 M and showed that the following process should also be considered in establishing the kinetics and mechanism of the title reaction under our experimental conditions:
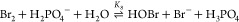
12with an equilibrium constant of *K*_8_ = 4.17 × 10^–7^ M^–1^. Consequently, the principle of detailed balancing^34^ provides an equilibrium constant of *K*_a1_ = *K*_7_/*K*_8_ = 0.0145 M for the first acid dissociation constant of phosphoric acid, resulting in the value of p*K*_a1_ = 1.84. Thus, if any small pH change is taken into consideration during the course of the title reaction, the acid dissociation process of phosphoric acid must be included with the above-mentioned p*K*_a1_ value not to violate the principle of detailed balancing, which is surprisingly not such an uncommon phenomenon in establishing the complex mechanism as pointed out recently by Stanbury and Hoffman.^35^

It is also well-known that in the presence of bromide ion bromine reacts to form tribromide ion in a rapidly established equilibrium:^36^
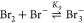
13It is a rather general belief (or better to say misbelief) that in the cases of the redox reactions of bromine, bromide ion inhibition may be encountered by the relative unreactivity of tribromide ion as compared to bromine. An example for that is the preliminary study of Dikshitulu and Babu^18^ on the title system. To clarify that this equilibrium alone is insufficient to explain quantitatively the bromide inhibition, eq 13 must be included fully in the proposed model with its well-established equilibrium constant. *K*_9_ was reported between the range of 16.8–19.2 M^–1^ depending on the experimental conditions.^37−39^ The value of 18.1 M^–1^ was chosen in this Article because that value was determined at the same experimental conditions (at *I* = 0.5 M ionic strength in the presence of phosphoric acid/dihydrogen-phosphate buffer).^39^

### Stoichiometry

The stoichiometry of the title reaction can be expressed by the following simple equation:
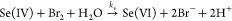
14Despite the simplicity of the stoichiometry, the kinetics of the title reaction was found to depict complex patterns such as H^+^- and bromide inhibition with changing formal kinetic orders suggested by the initial rate studies shown below.

### Initial Rate Studies

**Figure 1:**
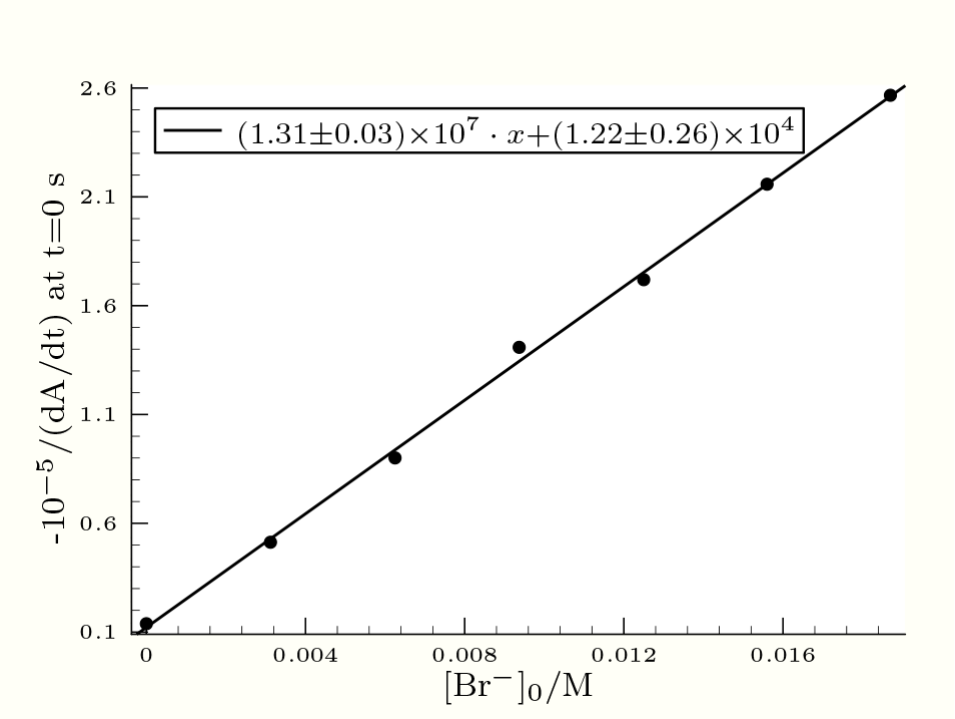
Plot of the inverse of the initial rate as a function of the initial bromide concentration. The conditions are as follows:  = 3.3 mM, [Se(IV)]_T,0_ = 0.49 mM, and pH = 1.25.

The log–log plots of the initial rates calculated from two series of experimental absorbance–time traces by varying the initial concentrations of selenium(IV) and bromine, respectively, meanwhile keeping other conditions unchanged, have clearly revealed that the formal kinetic orders of the reactants are equal purely to unity (see Figure S2). At the same time, the product bromide ion has a strong inhibitory effect as indicated in Figure 1, which agrees with the results published by Dikhsitulu and Babu.^18^

As it is clearly seen, the inverse of the initial rate plotted against the initial bromide concentration provided a perfect straight line with a notable intercept at a constant pH. Consequently, the rate of eq 14 should be formulated as follows:
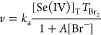
15where *A* can be considered as a function of [H^+^] and even that of other rate coefficients discussed later.

**Figure 2:**
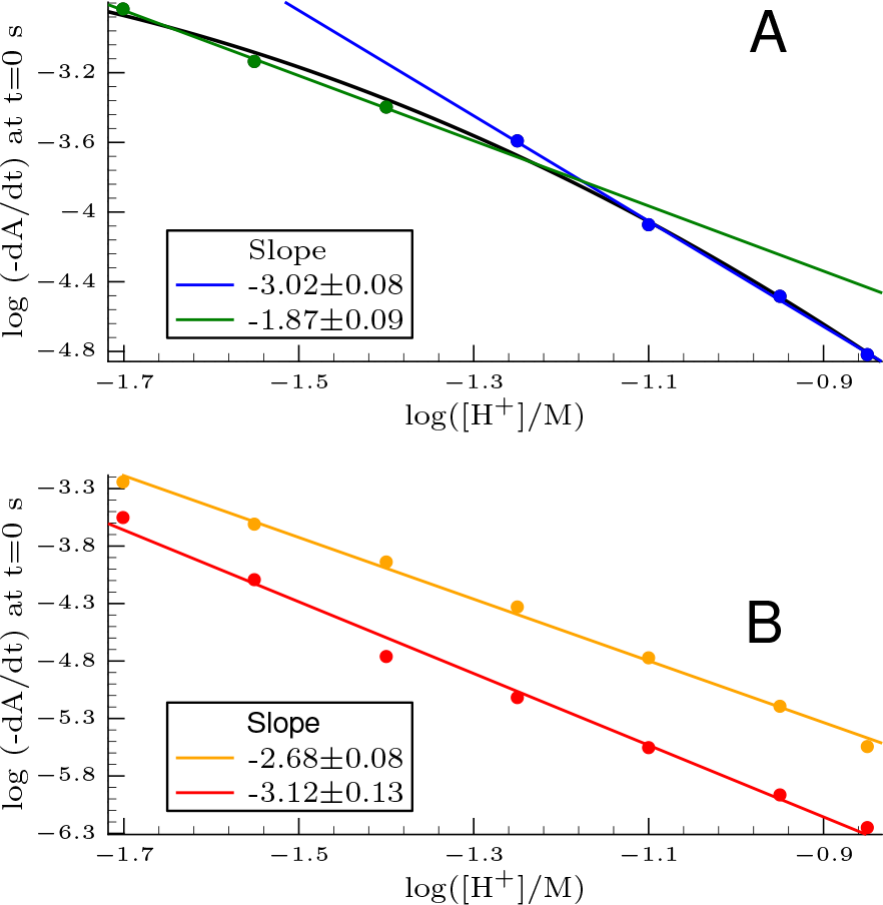
(A) Determination of the formal kinetic order of hydrogen ion in the absence of initially added bromide ion. Conditions are as follows: [Se(IV)]_T,0_ = 1.46 mM and  = 4.0 mM. Dots indicate the calculated initial rates at each pH value. The solid lines represented by the black color are the result of the least-square fit by a second-degree polynomial, while the blue and green lines represent the result of a linear regression to the four lowest and highest pH measured values. (B) Determination of the formal kinetic order of hydrogen ion in the presence of initially added bromide ion. The conditions are as follows: [Se(IV)]_T,0_ = 4.9 mM,  = 1.7 mM, and [Br^–^]_0_ = 9.4 mM (orange); [Se(IV)]_T,0_ = 4.9 mM,  = 1.1 mM, and [Br^–^]_0_ = 31.2 mM (red).

Furthermore, the effect of the hydrogen ion concentration on the reaction rate is even more complex as it is visualized in Figure 2. As it is seen in the absence of initially added bromide ion, the log–log plot of the initial rate versus hydrogen ion concentration significantly deviates from the straight line, indicating that the formal kinetic order of hydrogen ion markedly varies with the pH. The straight line fitted to the initial rates measured at lower pH values suggests a formal kinetic order of −3 resulting in a very strong inhibition, but this value increases to approximately −1.9. The situation becomes even more interesting in the presence of initially added bromide ion. At the lower bromide concentration, the log–log plot shows a sound straight line resulting in a formal kinetic order of −2.7 (see the orange curve in Figure 2B) that may further decrease by increasing the bromide concentration to −3.1 (red curve in Figure 2B). This complex dependence clearly suggests that *k*_a_ and *A* shown in eq 15 may have complex hydrogen dependence. Later, we shall show how this complex dependence may be interpreted by the proposed kinetic model.

### Quantitative Analysis of Previous Models

Our first probe for the quantitative description of the bromide inhibition was based on Dikshitulu and Babu’s report when eqs 13 and 14 were just considered in the kinetic model. *K*_9_ was set to 18.1 M^–1^ with the forward (*k*_9_) and reverse (*k*_–9_) rate coefficients adjusted to 1.81 × 10^9^ M^–1^ s^–1^ and 10^8^ s^–1^, respectively, and the apparent second-order rate coefficient (*k*_s_) was fitted to minimize the average deviation between the measured and calculated data in a series of kinetic runs where the pH, the initial bromine, and the initial selenium(IV) concentrations were all fixed and the initial bromide concentration was varied. Such a simplification makes it possible to focus just on the quantitative description of the bromide inhibition without considering the complex speciation of selenium(IV) and the bromine hydrolysis. We have determined *k*_s_ = 0.067 ± 0.003 M^–1^ s^–1^ by the least-square fit, but Figure S3 clearly indicates the inappropriateness of the model. We, therefore, concluded that the bromide inhibition cannot be described quantitatively by just supposing the unreactivity of tribromide ion over elementary bromine in the title reaction.

As a next step, we tried to use the kinetic model proposed by Liu et al.^25^ shown in eq 1 to describe quantitatively our data. Because this model is designed to describe the pH-dependence of the reaction, the speciation of selenium(IV) must be included in the model to check its validity. Therefore, for the calculations given below, we have used eqs 5–13 rapidly established equilibria with the previously mentioned *K*_1_–*K*_9_ equilibrium constants and eq 1 with their *k*_12_^″^, *k*_13_^″^, and *k*_22_^″^ rate constants shown in the Introduction. The average deviation for all of the kinetic curves (49 runs) measured was found to be an unacceptably high 0.0863 absorbance unit; therefore, we have tried to optimize the *k*_12_^″^, *k*_13_^″^, and *k*_22_^″^ rate coefficients by a nonlinear least-square fit. An almost reasonable average deviation was found (0.00745 absorbance unit), and the measured and calculated data are illustrated in Figure S4. This figure clearly shows that the model is not capable of describing correctly the bromine and the pH dependencies, where systematic errors may be seen. For the sake of correctness, however, it should also be emphasized that bromide inhibition seems to be described significantly better as compared to Dikshitulu and Babu’s report. As also expected, the result was quite insensitive for *k*_22_^″^, but *k*_12_^″^ = 795 M^–1^ s^–1^ and *k*_13_^″^ = 2.23 × 10^11^ M^–1^ s^–1^ could be calculated. The latter value, however, significantly exceeds the diffusion-controlled limit for second-order reactions in aqueous solutions; therefore, it cannot be realistic, from which we concluded that this model is also insufficient to describe quantitatively our kinetic data.

### Proposed Kinetic Model

From these preliminary calculations, one can easily conclude that it seems to be plausible to consider hypobromous acid as the kinetically active species toward selenium(IV). Therefore, as a first step to improve the kinetic model, we have taken into consideration the reactions of SeO_3_^2–^, HSeO_3_^–^, H_2_SeO_3_, H_4_(SeO_3_)_2_, ,  and  with hypobromous acid. Even though species  and  may only be present as low as 0.01% and 10^–8^% of the total selenium concentration, respectively, the very strong hydrogen ion inhibition observed in this system may require these reactions to be included as well. The complete kinetic model used in this calculation is represented in Table 1, and the average deviation between the measured and calculated data was found to be 0.0067 absorbance unit.

**Table 1:** Kinetic Model Involving HOBr as the Kinetically Active Species toward Selenium Species

step	reaction	rate laws	rate coefficients
(1)	SeO_3_^2–^ + H^+^ ⇌ HSeO_3_^–^	*k*_1_[SeO_3_^2–^][H^+^]	10^10^ M^–1^ s^–1^
		*k*_–1_[HSeO_3_^–^]	138 s^–1^
(2)	HSeO_3_^–^ + H^+^ *⇌* H_2_SeO_3_	*k*_2_[HSeO_3_^–^][H^+^]	10^10^ M^–1^ s^–1^
		*k*_–2_[H_2_SeO_3_]	4.9 × 10^7^ s^–1^
(3)	SeO_3_^2–^ + HSeO_3_^–^ ⇌ H(SeO_3_)_2_^3–^	*k*_3_[SeO_3_^2–^][HSeO_3_^–^]	10^10^ M^–1^ s^–1^
		*k*_–3_[H(SeO_3_)_2_^3–^]	3.89 × 10^9^ s^–1^
(4)	SeO_3_^2–^ + H_2_SeO_3_ ⇌ H_2_(SeO_3_)_2_^2–^	*k*_4_[SeO_3_^2–^][H_2_SeO_3_]	10^10^ M^–1^ s^–1^
		*k*_–4_[H_2_(SeO_3_)_2_^2–^]	1.1 × 10^4^ s^–1^
(5)	H^+^ + H_2_(SeO_3_)_2_^2–^ ⇌ H_3_(SeO_3_)_2_^–^	*k*_5_[H^+^][H_2_(SeO_3_)_2_^2–^]	10^10^ M^–1^ s^–1^
		*k*_–5_[H_3_(SeO_3_)_2_^–^]	1.32 × 10^7^ s^–1^
(6)	H^+^ + H_3_(SeO_3_)_2_^–^ ⇌ H_4_(SeO_3_)_2_	*k*_6_[H^+^][H_3_(SeO_3_)_2_^–^]	10^10^ M^–1^ s^–1^
		*k*_–6_[H_4_(SeO_3_)_2_]	5.75 × 10^7^ s^–1^
(7)	Br_2_ + H_2_O *⇌* HOBr + H^+^ + Br^–^	*k*_7_[Br_2_]	97 s^–1^
		*k*_–7_[HOBr][H^+^][Br^–^]	1.6 × 10^10^ M^–2^ s^–1^
(8)	Br_2_ + H_2_PO_4_^–^ + H_2_O *⇌* HOBr + Br^–^ + H_3_PO_4_	*k*_8_[Br_2_][H_2_PO_4_^–^]	10^3^ M^–1^ s^–1^
		*k*_–8_[HOBr][Br^–^][H_3_PO_4_]	2.4 × 10^9^ M^–2^ s^–1^
(9)	Br_2_ + Br^–^ *⇌* Br_3_^–^	*k*_9_[Br_2_][Br^–^]	1.81 × 10^9^ M^–1^ s^–1^
		*k*_–9_[Br_3_^–^]	10^8^ s^–1^
(10)	H_3_PO_4_ *⇌* H^+^ + H_2_PO_4_^–^	*k*_10_[H_3_PO_4_]	1.45 × 10^8^ s^–1^
		*k*_–10_[H^+^][H_2_PO_4_^–^]	10^10^ M^–1^ s^–1^
(I)	SeO_3_^2–^ + HOBr → SeO_4_^2–^ + Br^–^ + H^+^	*k*_I_[SeO_3_^2–^][HOBr]	(1.35 ± 0.06) × 10^11^ M^–1^ s^–1^ a
(II)	HSeO_3_^–^ + HOBr → SeO_4_^2–^ + Br^–^ + 2H^+^	*k*_II_[HSeO_3_^–^][HOBr]	7280 ± 450 M^–1^ s^–1^
(III)	H_2_SeO_3_ + HOBr → SeO_4_^2–^ + Br^–^ + 3H^+^	*k*_III_[H_2_SeO_3_][HOBr]	negligible
(IV)	H(SeO_3_)_2_^3–^ + 2HOBr → 2SeO_4_^2–^ + 2Br^–^ + 3H^+^	*k*_IV_[H(SeO_3_)_2_^3–^][HOBr]	(2.46 ± 0.26) × 10^13^ M^–1^ s^–1^ a
(V)	H_2_(SeO_3_)_2_^2–^ + 2HOBr → 2SeO_4_^2–^ + 2Br^–^ + 4H^+^	*k*_V_[H_2_(SeO_3_)_2_^2–^[HOBr]	(4.28 ± 0.22) × 10^6^ M^–1^ s^–1^
(VI)	H_3_(SeO_3_)_2_^–^ + 2HOBr → 2SeO_4_^2–^ + 2Br^–^ + 5H^+^	*k*_VI_[H_3_(SeO_3_)_2_^–^[HOBr]	negligible
(VII)	H_4_(SeO_3_)_2_ + 2HOBr → 2SeO_4_^2–^ + 2Br^–^ + 6H^+^	*k*_VII_[H_4_(SeO_3_)_2_][HOBr]	negligible

a Note that the *k*_I_ and *k*_IV_ values are unrealistic because they both exceed the diffusion control limit in the case of aqueous solutions.

The result is also illustrated in Figure S5. As it is seen, although the average deviation may seem to be almost sound, still one can easily notice systematic errors especially in the case of bromide dependence. In addition to that, two kinetic parameters (*k*_I_ and *k*_IV_, see Table 1) significantly exceed the diffusion control limit, and thus the present model cannot be realistic either. As a result, we concluded that the bromide inhibition found in this system can neither be described by supposing HOBr as the kinetically active bromine-containing species toward selenium(IV) nor be described by the unreactivity of . This phenomenon is strikingly similar to the case that we have already observed when studying the arsenous acid–iodine reaction.^22^ Therefore, as an analogy, in this system we may safely propose that one or more selenium(IV) species should rapidly be equilibrated with bromine to produce bromide ion by a bromonium ion transfer process to the substrate molecule as an initiating step.

#### Modeling Study by Supposing the Exclusive Reactivity of the Monomeric Selenium(IV) Species

The initiating step of the title reaction may be summarized as follows:
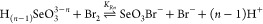
16where *n* can be 1, 2, and 3, when SeO_3_^2–^, HSeO_3_^–^, and H_2_SeO_3_ are supposed to be the kinetically active species, respectively. This step must be supplemented by the hydrolysis of SeO_3_Br^–^ to satisfy the stoichiometry of the reaction established:
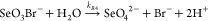
17where the rate term of eq 17 may be taken into consideration during the calculation to provide kinetically distinguished parallel routes as follows:
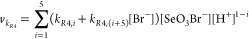
18This complicated rate term is justified by the initial rate studies. On the one hand, it is able to take into consideration the varying formal kinetic order of hydrogen ion in the absence and in the presence of initially added bromide ion (see the Initial Rate Studies subsection), and, on the other hand, it is also suitable to describe the notable intercept of the inverse of initial rate against the initial bromide concentration (see Figure 1), suggesting the presence of a bromide ion-independent term in the overall rate law (see eq 15). Of course, all of these *k*_*R*4,*i*_ and *k*_*R*4,(*i*+5)_ values were initially included at the beginning of the fitting procedure, but the number of the necessary but fitted kinetic parameters to give a correct quantitative description was reduced significantly to two by the end of these calculations. For the sake of completeness, we should emphasize that steps 1–10 indicated in Table 1 are also an obligatory part of these calculations to adjust the proper distribution of selenium(IV) and bromine species to obtain reliable kinetic parameters. Table 2 contains the results of all of the kinetic models, excluding the necessary equilibria, which were already shown in the upper part of Table 1, where the monomeric selenium(IV) species were considered as the kinetically active species in step-by-step fashion.

**Table 2:** Kinetic Parameters and the Average Deviation Obtained from Different Kinetic Models Supposing the Reactivity of the Monomeric Selenium Species^a^

parameters		model 1	model 2	model 3	model 4
*K*_*R*1_	*k*_*R*1_/M^–1^ s^–1^	(3.32 ± 0.05) × 10^7^	not used	not used	(4.33 ± 0.06) × 10^6^
	*k*_–*R*1_/M^–1^ s^–1^	10^7^	not used	not used	10^6^
*K*_*R*2_	*k*_*R*2_/M^–1^ s^–1^	not used	8.32 ± 0.10	not used	6.92 ± 0.09
	*k*_–*R*2_/M^–2^ s^–1^	not used	10^8^	not used	(1.16 ± 0.02) × 10^8^
*K*_*R*3_	*k*_*R*3_/M^–1^ s^–1^	not used	not used	1.21 ± 0.02	not sensitive
	*k*_–*R*3_/M^–3^ s^–1^	not used	not used	10^9^	not sensitive
	*k*_*R*4,1_	not sensitive	not sensitive	not sensitive	not sensitive
	*k*_*R*4,2_/M s^–1^	485 ± 8	257 ± 3	81.2 ± 0.3	361 ± 1
	*k*_*R*4,3_	not sensitive	not sensitive	not sensitive	not sensitive
	*k*_*R*4,4_	not sensitive	not sensitive	not sensitive	not sensitive
	*k*_*R*4,5_	not sensitive	not sensitive	not sensitive	not sensitive
	*k*_*R*4,6_	not sensitive	not sensitive	not sensitive	not sensitive
	*k*_*R*4,7_	not sensitive	not sensitive	not sensitive	not sensitive
	*k*_*R*4,8_	not sensitive	not sensitive	not sensitive	not sensitive
	*k*_*R*4,9_	not sensitive	not sensitive	not sensitive	not sensitive
	*k*_*R*4,10_/M^3^ s^–1^	(6.12 ± 0.31) × 10^–2^	(8.95 ± 0.35) × 10^–2^	(6.12 ± 0.25) × 10^–2^	0.100 ± 0.004
average deviation		0.0036	0.0032	0.0043	0.0031

a Wherever no standard deviation is indicated, the given parameter was fixed during the whole calculation process.

**Figure 3:**
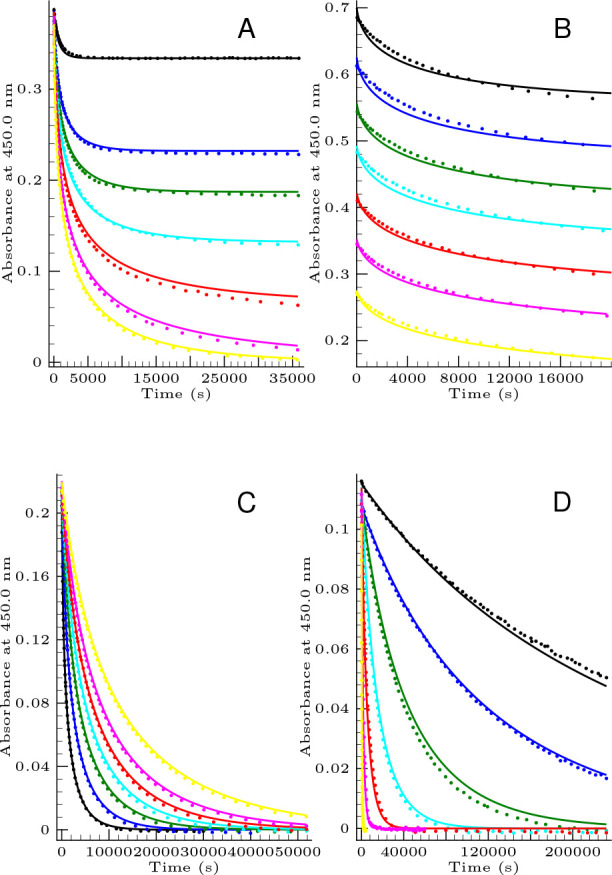
Experimental (symbols) and calculated (solid lines) absorbance–time traces at different experimental conditions. The calculated kinetic curves were obtained via model 2. (A)  = 3.7 mM, pH = 1.25, [Br^–^]_0_ = 0 mM. [Se(IV)]_*T*,0_/mM = 0.49 (black), 1.46 (blue), 1.95 (green), 2.44 (cyan), 3.17 (red), 4.14 (magenta), 4.88 (yellow). (B) [Se(IV)]_*T*,0_ = 1.46 mM, pH = 0.85, [Br^–^]_0_ = 0 mM. /mM = 6.7 (black), 6.0 (blue), 5.3 (green), 4.8 (cyan), 4.0 (red), 3.4 (magenta), 2.5 (yellow). (C)  = 2.1 mM, pH = 1.25, [Se(IV)]_*T*,0_ = 4.9 mM. [Br^–^]_0_/mM = 0 (black), 3.12 (blue), 6.25 (green), 9.37 (cyan), 12.5 (red), 15.6 (magenta), 21.9 (yellow). (D)  = 1.1 mM, [Se(IV)]_*T*,0_ = 4.9 mM [Br^–^]_0_ = 31.2 mM. pH = 0.85 (black), 0.95 (blue), 1.1 (green), 1.25 (cyan), 1.4 (red), 1.55 (magenta), 1.7 (yellow).

Comparison of the results of models 1–3 reveals that independently, whether SeO_3_^2–^, HSeO_3_^–^, or H_2_SeO_3_ is considered as the kinetically active species, the rate equation of the hydrolysis of SeO_3_Br^–^ may easily be simplified as follows:
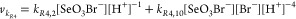
19The other most important consequence of these calculations is that supposing the exclusive reactivity of any of the monomeric selenium(IV) species shows a pretty sound description of the kinetic curves, but supposing H_2_SeO_3_ as the lone kinetically active species would lead to systematic deviations between the measured and calculated data. Therefore, we have concluded that under our experimental circumstance, H_2_SeO_3_ cannot be the kinetically active species. Third, in each kinetic model, we have found that the rate coefficient of the backward process of the initiating equilibrium should be large enough to treat SeO_3_Br^–^ as a short-lived intermediate. As a result, from our calculations, besides the absolute value of *k*_*R*2_ , actually the *k*_–*R*2_/*k*_*R*4,2_ and *k*_–*R*2_/*k*_*R*4,10_ ratios can be calculated, but not their absolute values. This phenomenon may be readily understood when discussing the overall rate law of the title reaction (see Analysis of the Proposed Kinetic Model subsection). If, however, SeO_3_^2–^ or HSeO_3_^–^ is considered as the lone reactive species, then the systematic deviation disappeared in both cases and a slightly better agreement was obtained when HSeO_3_^–^ was chosen as the reactive agent. Therefore, in the next calculation, both initiating equilibria were included considering the reactivity of HSeO_3_^–^ and SeO_3_^2–^. In this case, however, according to the principle of detailed balancing, the *K*_*R*1_/*K*_*R*2_ = *K*_1_ relationship must hold meaning in that *k*_–*R*2_ and *k*_*R*2_ cannot be independent from each other during the whole course of the calculation process.^34^ Thus, during the fitting procedure, while *k*_*R*2_ was fitted, for the value of *k*_–*R*2_, the *k*_–*R*2_ = *k*_*R*2_*K*_1_/*K*_*R*1_ relationship was established and the result can be seen in model 4. The average deviation was found to be just slightly better as compared to the previous cases, and all of the rate coefficients calculated are feasible making this kinetic model as the mathematically best option to propose. However, the almost negligible improvement in the average deviation obtained by using one more fitted kinetic parameter suggests that according to Ozzam’s razor model 2 already satisfies the simultaneous criterion of the quantitative agreement and the minimum number of fitted parameters to be used. Comparison of the measured and calculated curves indicated in Figure 3 just further supports this choice.

#### Modeling Study by Supposing the Exclusive Reactivity of Dimeric Selenium(IV) Species

To check whether the dimeric species might also play a decisive role in determining the kinetics of the title reaction, we have performed further calculations where only the dimer forms of selenium(IV) were taken into consideration to initiate the reaction. Therefore, the following initiating equilibrium was established between selenium-containing species and bromine:
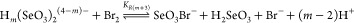
20where *m* may be varied from 2 to 4 to suppose , , and H_4_(SeO_3_)_2_, respectively, as the kinetically active species. One may easily miss the initiating equilibrium between  and bromine (*m* = 1); however, the concentration of this species in strongly acidic conditions is very low, which means that the title reaction cannot be driven via this pathway by realistic rate coefficients. Of course, eq 20 must be followed by eq 17 along with its rate equation (see eq 18). The results of these calculations are summarized in Table 3. For the sake of completeness, it should also be emphasized that steps 1–10 shown in Table 1 are also an inseparable part of the models in these calculation processes.

**Table 3:** Kinetic Parameters and the Average Deviation Obtained from Different Kinetic Models Supposing the Reactivity of the Dimeric Selenium Species^a^

parameters		model 5	model 6	model 7	model 8
*K*_*R*5_	*k*_*R*5_/M^–1^ s^–1^	(6.88 ± 0.24) × 10^4^	not used	not used	(4.98 ± 0.53) × 10^4^
	*k*_–*R*5_/M^–2^ s^–1^	10^8^	not used	not used	10^8^
*K*_*R*6_	*k*_*R*6_/M^–1^ s^–1^	not used	1340 ± 47	not used	302 ± 91
	*k*_–*R*6_/M^–3^ s^–1^	not used	2 × 10^9^	not used	(4.6 ± 1.2) × 10^8^
*K*_*R*7_	*k*_*R*7_/M^–1^ s^–1^	not used	not used	168 ± 6	not sensitive
	*k*_–*R*7_/M^–4^ s^–1^	not used	not used	4.2 × 10^9^	not sensitive
	*k*_*R*4,1_	not sensitive	not sensitive	not sensitive	not sensitive
	*k*_*R*4,2_/M s^–1^	(2.59 ± 0.13) × 10^–2^	(2.53 ± 0.14) × 10^–2^	(2.27 ± 0.13) × 10^–2^	(3.26 ± 0.30) × 10^–2^
	*k*_*R*4,3_/M^2^ s^–1^	(7.95 ± 0.33) × 10^–3^	(1.14 ± 0.02) × 10^–2^	(1.09 ± 0.02) × 10^–2^	(1.13 ± 0.02) × 10^–2^
	*k*_*R*4,4_	not sensitive	not sensitive	not sensitive	not sensitive
	*k*_*R*4,5_	not sensitive	not sensitive	not sensitive	not sensitive
	*k*_*R*4,6_	not sensitive	not sensitive	not sensitive	not sensitive
	*k*_*R*4,7_	not sensitive	not sensitive	not sensitive	not sensitive
	*k*_*R*4,8_	not sensitive	not sensitive	not sensitive	not sensitive
	*k*_*R*4,9_/M^2^ s^–1^	(6.75 ± 0.27) × 10^–3^	(9.19 ± 0.28) × 10^–3^	(1.05 ± 0.03) × 10^–2^	(9.17 ± 0.97) × 10^–3^
	*k*_*R*4,10_	not sensitive	not sensitive	not sensitive	not sensitive
average deviation		0.0047	0.0046	0.0046	0.0047

a Wherever no standard deviation is indicated, the given parameter was fixed during the whole calculation process.

From Table 3 three important conclusions may be drawn when inspecting models 5–7. First, the rate equation of the hydrolysis of SeO_3_Br^–^ (indicated by eq 18) may be simplified as follows:
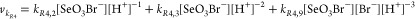
21Second, the average deviation obtained by considering the exclusive reactivity of , , and H_4_(SeO_3_)_2_ leads to almost the same, but a notably higher average deviation as compared to that of the previous models focusing on the exclusive reactivity of monomeric species. Third, even though to reach this average deviation requires one more parameter to be fitted, the results obtained were not perfect, and systematic deviation may be observed between the experimental and calculated data especially at the series belonging to the bromide dependence at middle pH’s and to the selenite dependence. As a result, these calculations clearly showed that supposing just one kinetically active dimeric selenium(IV) species is not enough to describe quantitatively the measured kinetic traces. Therefore, in the next calculation procedure, we have tried to use multiple dimeric Se(IV) species as kinetically active reactants. In this case, however, according to the principle of detailed balancing,^34^ the following equations must necessarily hold: *K*_*R*5_/*K*_*R*6_ = *K*_5_ and *K*_*R*5_/*K*_*R*7_ = *K*_5_*K*_6_, which means that *k*_–*R*6_ = *k*_*R*6_*k*_*R*5_*k*_5_/(*k*_–5_*k*_–*R*5_) and *k*_–*R*7_ = *k*_*R*7_*k*_*R*5_*k*_5_*k*_6_/(*k*_–5_*k*_–6_*k*_–*R*5_) equalities should always be fulfilled during the calculation process. Using these conditions, the simultaneous evaluation of the kinetic curves clearly revealed that *K*_*R*7_ equilibrium can safely be removed from the model, and the values of the kinetic parameters obtained are given in the final column of Table 3 indicated by model 8. From these data, one can easily conclude that the quality of the fit cannot be improved by inclusion of the reactivity of more than one dimeric species (see the average deviation), and still the systematic deviation between the measured and calculated data remained almost the same (see Figure S6). Moreover, the average deviation obtained from models 5–8 is notably higher as compared to those calculated by models 1–4; thus it appears to be evident that the kinetically active species under our experimental conditions are the monomeric forms of selenite ion. The only remaining question is whether any of the dimeric forms of selenite might play any role in determining the kinetics of the title reaction along with the monomeric form or not.

#### Modeling Study to Consider the Combined Reactivity of the Monomeric and the Dimeric Selenium(IV) Species

To answer the question raised in the previous subsection, additional calculation processes were carried out, where the reactivities of SeO_3_^2–^, HSeO_3_^–^, , and  were all considered. As a result, the title reaction was supposed to be initiated via *K*_*R*1_, *K*_*R*2_, *K*_*R*5_, and *K*_*R*6_ equilibria. It should be noted that *K*_*R*7_ can readily be eliminated on the basis of the previous calculations mentioned in the previous subsections. According to the principle of detailed balancing,^34^ the following relationships were per se introduced among the equilibrium constants: *K*_*R*1_/*K*_*R*2_ = *K*_1_, *K*_*R*1_/*K*_*R*5_ = *K*_4_, and *K*_*R*1_/*K*_*R*6_ = *K*_5_*K*_4_ establishing the equations between the corresponding rate coefficients necessary to hold, such as *k*_–*R*2_ = *k*_*R*2_*k*_–*R*1_*k*_1_/(*k*_*R*1_*k*_–1_), *k*_–*R*5_ = *k*_*R*5_*k*_–*R*1_*k*_4_/(*k*_*R*1_*k*_–4_), and *k*_–*R*6_ = *k*_*R*6_*k*_–*R*1_*k*_5_*k*_4_/(*k*_*R*1_*k*_–5_*k*_–4_), during the course of the whole calculation process. In other words, it means if *k*_–*R*1_ is fixed by any reason and the equilibria belonging to the *K*_*R*1_, *K*_*R*2_, *K*_*R*5_, and *K*_*R*6_ constants are involved in the chemical model, then *k*_–*R*2_, *k*_–*R*5_, and *k*_–*R*6_ cannot be set arbitrarily; they have to be necessarily calculated from the above-mentioned equations, otherwise the principle of detailed balancing is violated. In the kinetic model, these equilibria must be followed by the hydrolysis of SeO_3_Br^–^ (see eq 17) by its corresponding rate law indicated by eq 18. These calculations have revealed that the average deviation obtained by using model 4 cannot be decreased further even if all of the above-mentioned selenium species are included as kinetically active reactants. As a result, according to Ozzam’s razor, we propose the simplest kinetic model (model 2 indicated in Table 2) to describe our kinetic data under the experimental conditions applied. It should, however, be emphasized that it does not rule out that significantly different experimental conditions may lead to a different conclusion where another form of selenium(IV) species is both mathematically and chemically a better choice to drive the reaction. The slightly better average deviation when HSeO_3_^–^ and SeO_3_^2–^ were simultaneously used to drive the reaction (similar final result was also obtained when HSeO_3_^–^ and  were both considered as kinetically active species in the kinetic model) may straightforwardly suggest that application of lower pH’s or higher total selenium(IV) concentration might require other form(s) to be selected as kinetically active forms of selenium(IV).

#### Analysis of the Proposed Kinetic Model

Table 4 comprises all of the processes, their rate laws, and the fixed and fitted rate coefficients used to describe our experimental data. It directly allows us, first, to derive the overall rate law from this model, second, to compare it with the experimentally established rate law indicated by eq 15, and, third, also to interpret the unusual changes in the formal kinetic order pH and bromide ion.

**Table 4:** Proposed Kinetic Model^a^

step	reaction	rate laws	rate coefficients
(1)	SeO_3_^2–^ + H^+^ ⇌ HSeO_3_^–^	*k*_1_[SeO_3_^2–^][H^+^]	10^10^ M^–1^ s^–1^
		*k*_–1_[HSeO_3_^–^]	138 s^–1^
(2)	HSeO_3_^–^ + H^+^ *⇌* H_2_SeO_3_	*k*_2_[HSeO_3_^–^][H^+^]	10^10^ M^–1^ s^–1^
		*k*_–2_[H_2_SeO_3_]	4.9 × 10^7^ s^–1^
(3)	SeO_3_^2–^ + HSeO_3_^–^ ⇌ H(SeO_3_)_2_^3–^	*k*_3_[SeO_3_^2–^][HSeO_3_^–^]	10^10^ M^–1^ s^–1^
		*k*_–3_[H(SeO_3_)_2_^3–^]	3.89 × 10^9^ s^–1^
(4)	SeO_3_^2–^ + H_2_SeO_3_ ⇌ H_2_(SeO_3_)_2_^2–^	*k*_4_[SeO_3_^2–^][H_2_SeO_3_]	10^10^ M^–1^ s^–1^
		*k*_–4_[H_2_(SeO_3_)_2_^2–^]	1.1 × 10^4^ s^–1^
(5)	H^+^ + H_2_(SeO_3_)_2_^2–^ ⇌ H_3_(SeO_3_)_2_^–^	*k*_5_[H^+^][H_2_(SeO_3_)_2_^2–^]	10^10^ M^–1^ s^–1^
		*k*_–5_[H_3_(SeO_3_)_2_^–^]	1.32 × 10^7^ s^–1^
(6)	H^+^ + H_3_(SeO_3_)_2_^–^ ⇌ H_4_(SeO_3_)_2_	*k*_6_[H^+^][H_3_(SeO_3_)_2_^–^]	10^10^ M^–1^ s^–1^
		*k*_–6_[H_4_(SeO_3_)_2_]	5.75 × 10^7^ s^–1^
(7)	Br_2_ + H_2_O *⇌* HOBr + H^+^ + Br^–^	*k*_7_[Br_2_]	97 s^–1^
		*k*_–7_[HOBr][H^+^][Br^–^]	1.6 × 10^10^ M^–2^s^–1^
(8)	Br_2_ + H_2_PO_4_^–^ + H_2_O *⇌* HOBr + Br^–^ + H_3_PO_4_	*k*_8_[Br_2_][H_2_PO_4_^–^]	10^3^ M^–1^ s^–1^
		*k*_–8_[HOBr][Br^–^][H_3_PO_4_]	2.4 × 10^9^ M^–2^ s^–1^
(9)	Br_2_ + Br^–^ *⇌* Br_3_^–^	*k*_9_[Br_2_][Br^–^]	1.81 × 10^9^ M^–1^ s^–1^
		*k*_–9_[Br_3_^–^]	10^8^ s^–1^
(10)	H_3_PO_4_ *⇌* H^+^ + H_2_PO_4_^–^	*k*_10_[H_3_PO_4_]	1.45 × 10^8^ s^–1^
		*k*_–10_[H^+^][H_2_PO_4_^–^]	10^10^ M^–1^ s^–1^
(R2)	HSeO_3_^–^ + Br_2_ *⇌* SeO_3_Br^–^ + Br^–^ + H^+^	*k*_*R*2_[HSeO_3_^–^][Br_2_]	8.32 ± 0.10 M^–1^ s^–1^
		*k*_–*R*2_[SeO_3_Br^–^][Br^–^][H^+^]	10^8^ M^–2^ s^–1^
(R4)	SeO_3_Br^–^ + H_2_O → SeO_4_^2–^ + Br^–^ + 2H^+^	*k*_*R*4,2_[SeO_3_Br^–^][H^+^]^−1^	257 ± 3 M s^–1^
		*k*_*R*4,10_[SeO_3_Br^–^][Br^–^][H^+^]^−4^	(8.95 ± 0.35) × 10^–2^ M^3^ s^–1^

a If no standard deviation is indicated, then the given parameter is fixed during the whole calculation process.

The total selenium(IV) concentration ([Se(IV)]_T_) can be expressed by the following equation:
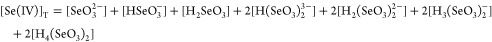
22Using the definition of equilibrium constants *K*_1_–*K*_6_, this equation may be rearranged as follows:
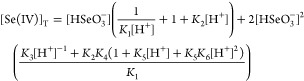
23The second-degree polynomial expression may easily be solved for [HSeO_3_^–^], and the positive root is given as
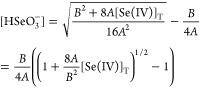
24where
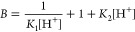
25and
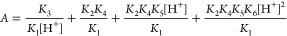
26

Because under our experimental conditions the 8*A*[Se(IV)]/*B*^2^ < 1 inequality holds, eq 24 may easily be approximated as follows:
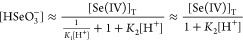
27This equation is also supported by the fact that under our experimental conditions, more than 93% of the selenium(IV) species is present in the form of HSeO_3_^–^ and H_2_SeO_3_ (see Figure S1). Applying the steady-state approximation for species SeO_3_Br^–^
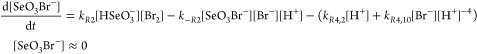
28leads to
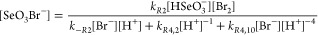
29Because
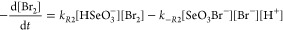
30then substitution of eq 29 into eq 30 followed by some algebraic rearrangements leads to the following expression:
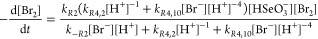
31Substitution of eq 27 and  into eq 31 followed by eliminating the negligible terms in the denominator results in the following expression:
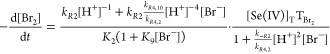
32Comparing eq 32 with eq 15 reveals that
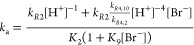
33and if the pH ≤ 1.25 as well as [Br^–^]_0_ < 0.02 M inequalities are fulfilled, then *k*_a_ is fairly constant, which soundly explains why the inverse of the measured initial rate was found to be perfectly linear as a function of bromide concentration (see Figure 1). In addition, it also provides the explicit [H^+^] and rate coefficient dependence of *A* shown in eq 15. Last, eq 32 clearly explains as well why the absolute value of *k*_*R*2_ along with the *k*_–*R*2_/*k*_*R*4,2_ and *k*_–*R*2_/*k*_*R*4,10_ ratios can only be determined from our experimental data.

### Application of the Kinetic Model in the Extended pH Range

**Figure 4:**
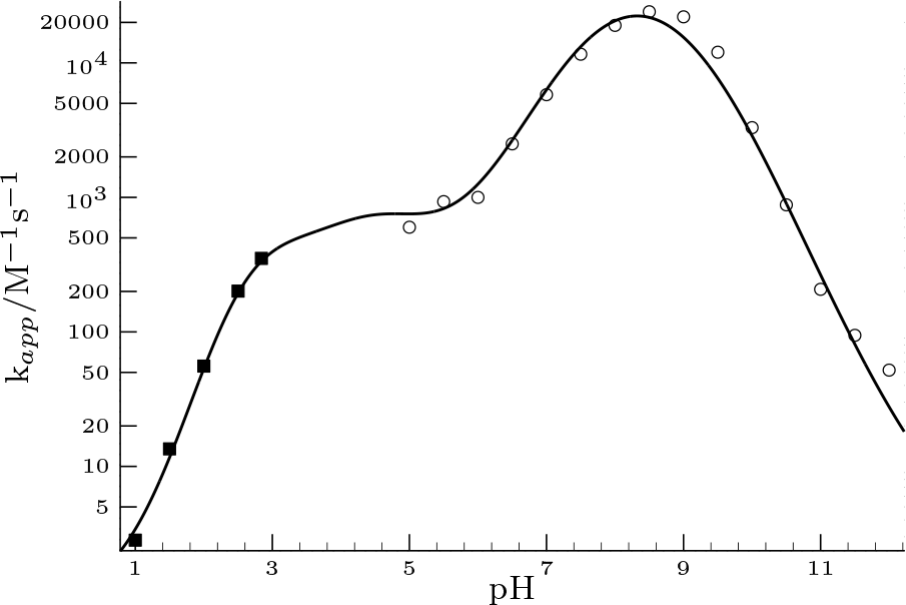
Dependence of the apparent second-order rate coefficient as a function of pH by merging model 2 presented here and the modified model published by Liu et al.^25^ [Adapted in part with permission from Liu et al./Water Research. Copyright 2019 Elsevier.] The “○” correspond to the *k*_app_ values found in the Supporting Information of ref (25), while the “■” were obtained from our measurements.

Admittedly, the model proposed here is working properly under strongly acidic condition. Therefore, we have extended the kinetic model presented in Table 4 with two additional reactions shown in Table 1 as steps I and II proposed by Liu et al.^25^ to extend the pH range where the new model predicts *k*_app_ values soundly. We have thus adopted their specific rate coefficients reported therein as *k*_12_^″^ = 2300 M^–1^ s^–1^ and *k*_13_^″^ = 74 000 M^–1^ s^–1^, but neglected *k*_22_^″^. The reason for this omission has already been shown in the Introduction. The apparent rate coefficient (*k*_app_) may be defined as
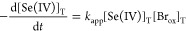
34where [Br_ox_]_T_ = [Br_2_] + [Br_3_^–^] + [HOBr] + [OBr^–^], and [Se(IV)]_T_ stands for the total selenium(IV) concentration given in eq 22. Figure S7 clearly shows that the calculated *k*_app_ values are clearly overestimated at lower pH’s by inclusion of steps I and II with the given rate coefficients. Another indication that *k*_12_^″^ and *k*_13_^″^ are overestimated may be seen in Figure 2b of ref (25). Around pH = 5–6 HOBr and HSeO_3_^–^ are exclusively present as selenium- and bromine-containing species, and thus *k*_app_ ≈ *k*_13_^″^ should hold. Instead of that, the Supporting Information of ref (25) shows significantly lower measured values ranging between 600–1000 M^–1^ s^–1^. From their measurements, we have estimated *k*_12_^″^ = 700 M^–1^ s^–1^. A quite similar argument may also be mentioned for *k*_13_^″^; thus we have decreased it for 44 000 M^–1^ s^–1^ and recalculated *k*_app_ values as a function of pH represented in Figure 4.

To extend our previous measurements, we have performed two more experiments to determine the *k*_app_ values at pH = 2.5 and at pH = 2.84 as well by using the same experimental conditions. As it is seen, the extended model almost perfectly agrees with the trend of *k*_app_ values as a function of a wide pH range validating the usefulness of the present model for further calculations.

## Conclusion

In this Article, we have clearly demonstrated that the initial step of the selenite–bromine reaction at strongly acidic conditions is a bromonium transfer process leading to an equilibrium between the bromine molecule and selenium(IV) species to form bromoselenate (SeO_3_Br^–^) and bromide ions followed by the hydrolysis of this short-lived intermediate. Even though hypobromous acid is also capable of a bromonium ion transfer process shown by Troy and Margerum^40^ and considering this species as the reactive form of a bromine-containing compound instead of bromine also accounts for the inhibitory effect of bromide ion; this conceivable route can easily be ruled out under strongly acidic conditions by the fact that the rate coefficient of the selenite–hypobromous acid reaction would significantly exceed the diffusion-controlled limit of second-order reactions in aqueous solution to describe the measured kinetic traces simultaneously. Furthermore, we have shown, providing the results of numerous alternative kinetic models, that even though several selenium species are present under our experimental circumstances, the kinetically active form can safely be assumed as HSeO_3_^–^. Any further trials to include more than one selenium species as kinetically active reactants failed to show significant improvement in the average deviation between our measured and calculated data.

A more important consequence of our present work is that the kinetic model working at strongly acidic conditions can readily be extended by the direct reactions of the monomeric forms of selenium(IV) species with hypobromous acid proposed by Liu et al.^25^ with modified rate coefficients. The overlooked effect of the pathway shown here resulted in a notable overestimation of these rate coefficients calculated previously (*k*_12_^″^ and *k*_13_^″^), but combining the chemical information obtained at strongly acidic conditions and reported in the pH range of 5–12 by Liu et al.^25^ made it possible to refine these parameters. The kinetic model that we developed is thus capable of providing a useful and more accurate tool working already in the range of pH = 1–13 to predict the fate of selenium species during water treatment processes.

Last, our present work still provides a general but straightforward procedure of how to avoid violation of the fundamental rule of detailed balancing when more than one kinetically active form of rapidly equilibrated species should be taken into consideration to obtain meaningful kinetic models. Avoiding violations of the principle of detailed balancing is much more important than one might think. Even in environmentally relevant kinetic problems, numerous incorrect examples were recently listed in ref (41). Where, if not here influencing the processes taking place in our environment, would these scientific problems be calmly challenged?

## Supporting Information Available

Table containing the stability constants for selenium species, figures indicating the relative amount of selenium species at strongly acidic conditions, the formal kinetic orders of the reactants, and the inappropriateness of the models discussed (PDF)
