# Supplementary material for: Kinetics and Mechanism of Selenium(IV) Oxidation by Aqueous Bromine Solution
Source: ACS Omega. 2023 Apr 21;8(17):15769–80. doi: 10.1021/acsomega.3c01497 (PMC10157880; doi:10.1021/acsomega.3c01497)
Supplement: Supplementary file 1 — ao3c01497_si_001.pdf [file ao3c01497_si_001.pdf]

Supporting Information for Kinetics and Mechanism of Selenium(IV) Oxidation by Aqueous Bromine Solution

György Csekő, Boglárka Nyitrai, and Attila K. Horváth*

Department of General and Inorganic Chemistry, Faculty of Sciences, University of Pécs, Ifjúság útja 6., H-7624 Pécs, Hungary

E-mail:

Table S1: Upper part: Formation constants of H_qL_p species determined by Barcza and Sillén at different ionic strengths adjusted by sodium perchlorate. The values at I = 0.5 M were estimated from the average of the ones reported at 0.3 M and 1.0 M ionic strengths. Lower part: Equilibria and their corresponding equilibrium constants derived from the formation constants estimated at I=0.5 M sodium perchlorate medium used in the fitting procedure.

(p,q)	Equilibrium	β_{pq}	logβ_{pq} (I=1 M)	logβ_{pq} (I=0.3 M)	logβ_{pq} (I=0.5 M)
(1,1)	$H+L \rightleftharpoons HL$	$\beta_{11}=\frac{[HL]}{[H][L]}$	7.78	7.94	7.86
(1,2)	$2H+L \rightleftharpoons H_2L$	$\beta_{12}=\frac{[H_2L]}{[H]^2[L]}$	10.05	10.29	10.17
(2,1)	$H+2L \rightleftharpoons HL_2$	$\beta_{21}=\frac{[HL_2]}{[H][L]^2}$	8.01	8.54	8.27
(2,2)	$2H+2L \rightleftharpoons H_2L_2$	$\beta_{22}=\frac{[H_2L_2]}{[H]^2[L]^2}$	15.73	16.52	16.13
(2,3)	$3H+2L \rightleftharpoons H_3L_2$	$\beta_{23}=\frac{[H_3L_2]}{[H]^3[L]^2}$	18.7	19.32	19.01
(2,4)	$4H+2L \rightleftharpoons H_4L_2$	$\beta_{24}=\frac{[H_4L_2]}{[H]^4[L]^2}$	20.8	21.71	21.25

Equilibria used in the fitting procedure		Equilibrium constant
(R1)	$H+L \rightleftharpoons HL$	$\log K_1=\log\beta_{11}=7.86$
(R2)	$H+HL \rightleftharpoons H_2L$	$\log K_2=\log\beta_{12}-\log\beta_{11}=2.31$
(R3)	$L+HL \rightleftharpoons HL_2$	$\log K_3=\log\beta_{21}-\log\beta_{11}=0.41$
(R4)	$L+H_2L \rightleftharpoons H_2L_2$	$\log K_4=\log\beta_{22}-\log\beta_{12}=5.96$
(R5)	$H+H_2L_2 \rightleftharpoons H_3L_2$	$\log K_5=\log\beta_{23}-\log\beta_{22}=2.88$
(R6)	$H+H_3L_2 \rightleftharpoons H_4L_2$	$\log K_6=\log\beta_{24}-\log\beta_{23}=2.24$

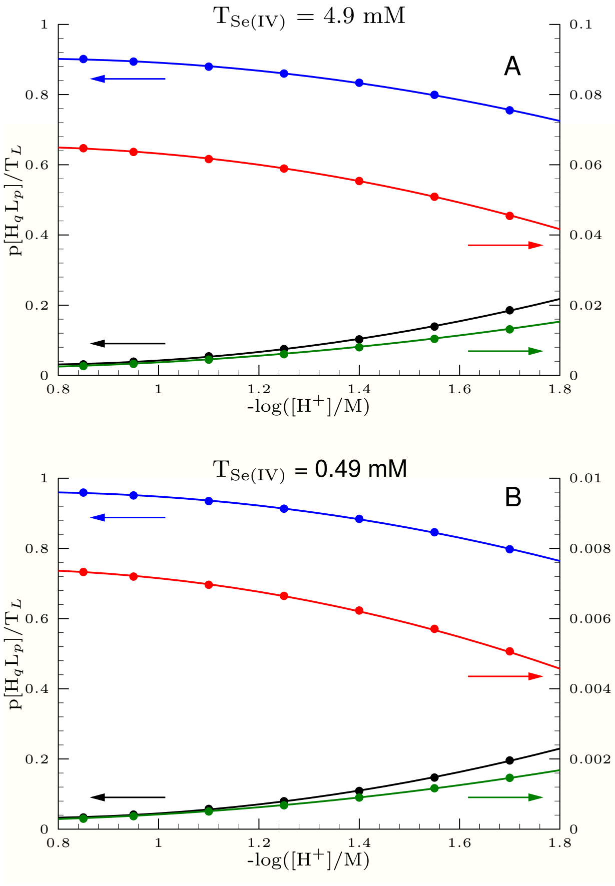

Figure S1: Distribution diagram of selenium containing species at the highest (A) and at the lowest (B) initial total selenite concentration. Species: H_2SeO_3 (blue); $HSeO_3^-$ (black); $H_4(SeO_3)_2$ (red); $H_3(SeO_3)_2^-$ (green). Note that the left and right Y-axis belongs to the monomer and dimer species, respectively.

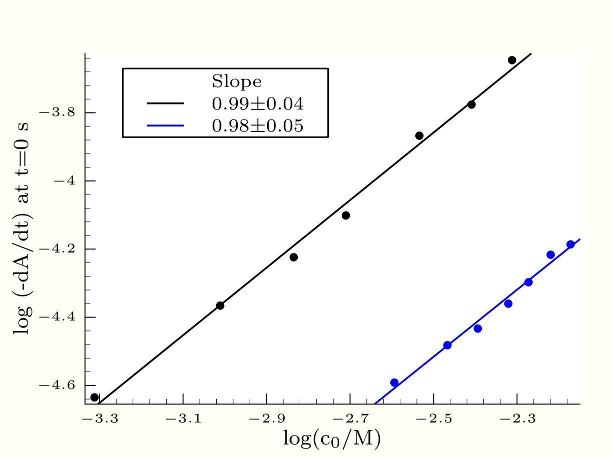

Figure S2: Determination of the formal kinetic orders of selenium(IV) (black) and bromine (blue) from initial rate studies at pH = 0.85 in the abscence of initially added bromide ion. c_0 corresponds to the initial concentration of selenium(IV) and bromine, respectively. Other conditions are as follows: $T^0_{Br_2}$ = 5.15 mM (black) and $T^0_{Se(IV)}$ = 1.46 mM (blue).

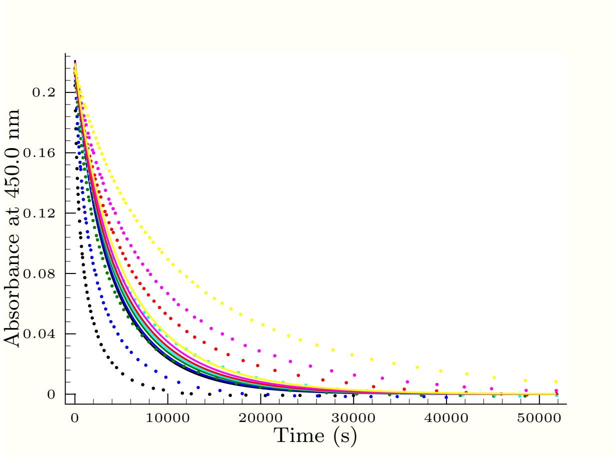

Figure S3: Measured (symbols) and calculated (solid lines) absorbance–times traces at pH = 1.25; $[Se(IV)]_0$ = 4.9 mM; $T^0_{Br_2}$ = 2.1 mM. $[Br^-]_0$/mM = 0 (black), 3.1 (blue), 6.25 (green), 9.4 (cyan), 12.5 (red), 15.6 (magenta), 21.9 (yellow). The solid lines was calculated by using eqs. (13) and (14) with rate constant of $k_s = 0.068$ $M^{-1}s^{-1}$ and equilibrium constant of K_9 =18.1 M^{-1}. The average deviation 0.026 a.u. clearly indicates the deficiency of Dikshitulu and Babu's model to describe the bromide inhibition quantitatively.

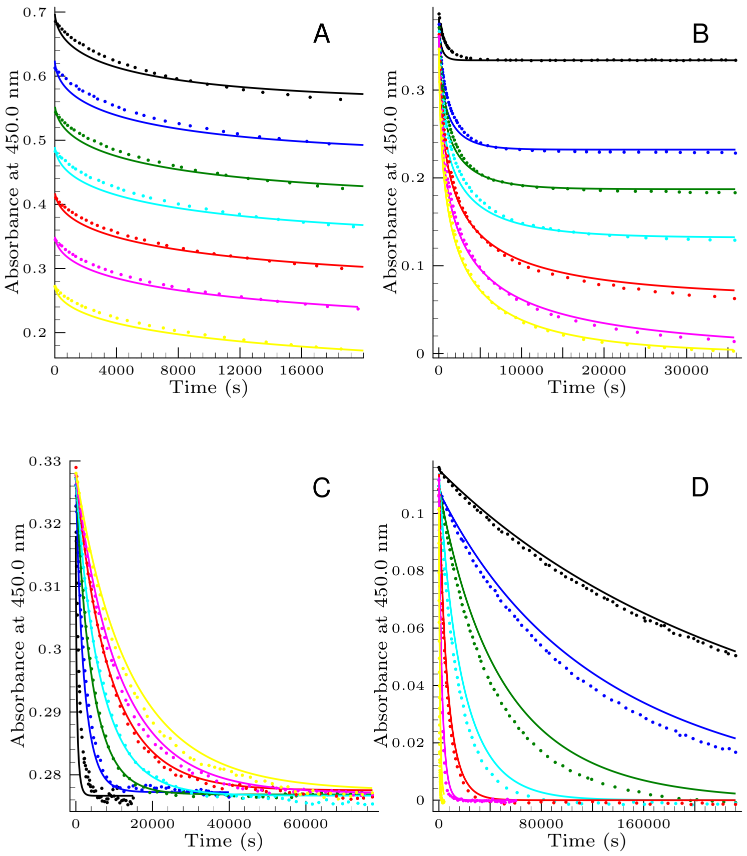

Figure S4: The result of the simultaneous evaluation of all the measured kinetic traces by the kinetic model proposed by Liu et al. The values for K_1–K_9 can be found in the original manuscript, k''_{12} and $''_{13}$ were found to be 795 $M^{-1}s^{-1}$ and 2.23×10^{11} $M^{-1}s^{-1}$, respectively. The latter value is unrealistic because it hits the diffusion controlled limit for second-order reactions in aqueous solutions. Measured and calculated data are illustrated by symbols and solid lines, respectively. The conditions are as follows: (A) $[Se(IV)]_0 = 1.46$ mM; pH = 0.85; $[Br^-]_0 = 0$ mM; $T^0_{Br_2}$/mM = 6.7 (black), 6.0 (blue), 5.3 (green), 4.8 (cyan), 4.0 (red), 3.4 (magenta), 2.5 (yellow); (B) $T^0_{Br_2} = 3.7$ mM; pH = 1.25; $[Br^-]_0 = 0$ mM; $[Se(IV)]_0$/mM = 0.49 (black), 1.46 (blue), 1.95 (green), 2.44 (cyan), 3.17 (red), 4.14 (magenta), 4.88 (yellow); (C) $T^0_{Br_2} = 3.3$ mM; pH = 1.25; $[Se(IV)]_0 = 0.49$ mM; $[Br^-]_0$/mM = 0.0 (black), 3.1 (blue), 6.25 (green), 9.37 (cyan), 12.5 (red), 15.6 (magenta), 18.7 (yellow); (D) $T^0_{Br_2} = 1.1$ mM; $[Se(IV)]_0 = 4.9$; $[Br^-]_0 = 31.2$ mM; pH = 0.85 (black), 0.95 (blue), 1.1 (green), 1.25 (cyan), 1.4 (red), 1.55 (magenta), 1.70 (yellow).

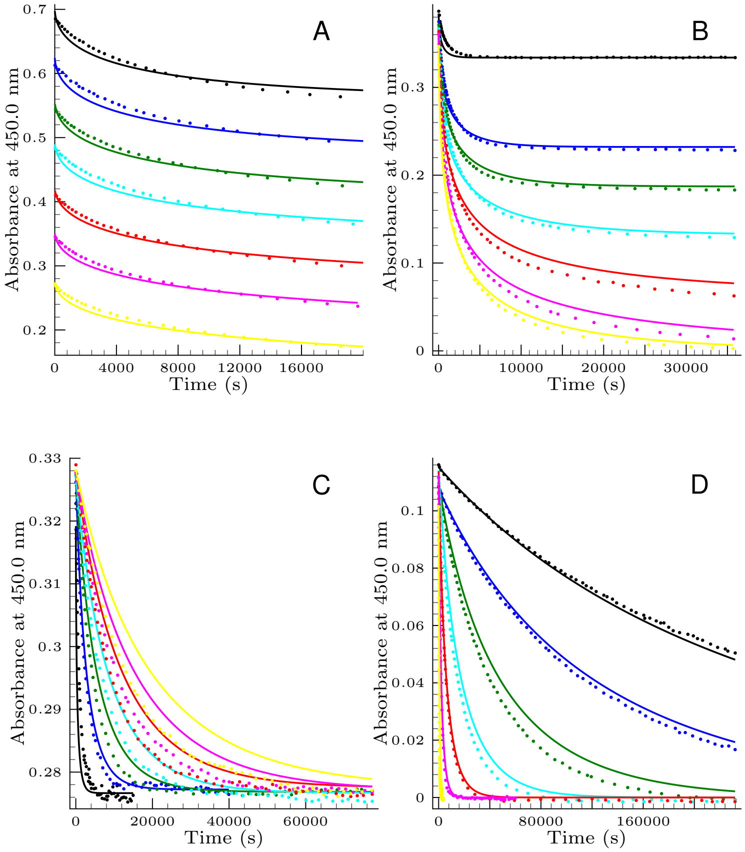

Figure S5: The result of the simultaneous evaluation of all the measured kinetic traces by the kinetic model indicated in Table 1 of the manuscript. Note that two kinetic parameters obtained significantly hit the diffusion control limit. Measured and calculated data are illustrated by symbols and solid lines, respectively. The conditions are as follows: (A) $[Se(IV)]_0 = 1.46$ mM; pH = 0.85; $[Br^-]_0 = 0$ mM; $T^0_{Br_2}$/mM = 6.7 (black), 6.0 (blue), 5.3 (green), 4.8 (cyan), 4.0 (red), 3.4 (magenta), 2.5 (yellow); (B) $T^0_{Br_2} = 3.7$ mM; pH = 1.25; $[Br^-]_0 = 0$ mM; $[Se(IV)]_0$/mM = 0.49 (black), 1.46 (blue), 1.95 (green), 2.44 (cyan), 3.17 (red), 4.14 (magenta), 4.88 (yellow); (C) $T^0_{Br_2} = 3.3$ mM; pH = 1.25; $[Se(IV)]_0 = 0.49$ mM; $[Br^-]_0$/mM = 0.0 (black), 3.1 (blue), 6.25 (green), 9.37 (cyan), 12.5 (red), 15.6 (magenta), 18.7 (yellow); (D) $T^0_{Br_2} = 1.1$ mM; $[Se(IV)]_0 = 4.9$; $[Br^-]_0 = 31.2$ mM; pH = 0.85 (black), 0.95 (blue), 1.1 (green), 1.25 (cyan), 1.4 (red), 1.55 (magenta), 1.70 (yellow).

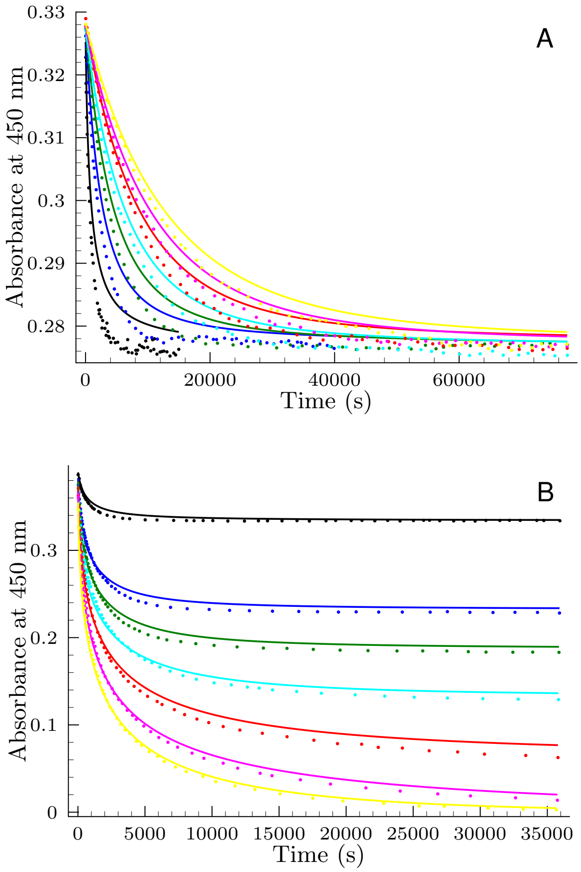

Figure S6: Result of the simultaneous evaluation of the measured kinetic curves by using kinetic model 8 in Table 3. Measured and calculated data are illustrated by symbols and solid lines, respectively. The conditions are as follows: (A) $[\mathrm{Se(IV)}]_0$ = 0.49 mM, $\mathrm{T}^0_{\mathrm{Br}_2}$ = 3.3 mM, pH = 1.25. $[\mathrm{Br}^-]_0$/mM = 0.0 (black), 3.1 (blue), 6.25 (green), 9.37 (cyan), 12.5 (red), 15.6 (magenta), 18.7 (yellow). (B) $\mathrm{T}^0_{\mathrm{Br}_2}$ = 3.7 mM, $[\mathrm{Br}^-]_0$ = 0 mM, pH = 1.25, $[\mathrm{Se(IV)}]_0$/mM = 0.49 (black), 1.46 (blue), 1.95 (green), 2.44 (cyan), 3.17 (red), 4.14 (magenta), 4.88 (yellow).

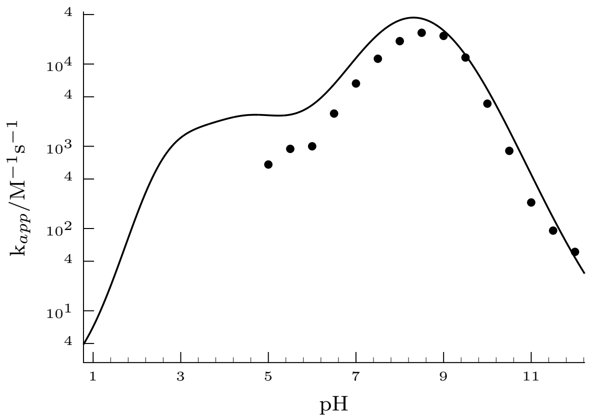

Figure S7: Calculated second-order rate coefficients (solid line) as a function of pH obtained by merging the kinetic models suggested by Liu et al. (see: references) and the one presented here as a function of pH. The symbols correspond to the measured values reported in the supporting information of Liu et al. paper.
